# Enhancing healthcare through ontology: a systematic review of challenges and future directions

**DOI:** 10.3389/frai.2026.1872024

**Published:** 2026-08-19

**Authors:** U. Priyadharshini, R. Vijayan

**Affiliations:** 1 School of Advanced Sciences, Vellore Institute of Technology (VIT), Vellore, India; 2 School of Computer Science Engineering and Information Systems, Vellore Institute of Technology (VIT), Vellore, India

**Keywords:** clinical knowledge representation, healthcare ontology, medical data interoperability, ontology in personalized medicine, semantic data integration

## Abstract

This study explores the role of ontology in healthcare by surveying numerous research articles to provide a comprehensive overview of its applications, benefits, and challenges. Ontologies, which enable structured representation and integration of complex healthcare knowledge, have been increasingly employed to enhance data interoperability, improve clinical decision making, and support personalized medicine. Despite their potential, the development and implementation of ontologies in healthcare face challenges, including issues with data consistency, interoperability across systems, and adaptation to rapidly evolving medical knowledge. Using the Preferred Reporting Items for Systematic Reviews and Meta-Analyses (PRISMA) model, we systematically identified, screened, and reviewed relevant studies. This model enabled a rigorous process for article selection, ensuring inclusion of high-quality research that addressed key themes in ontology-based healthcare systems. The survey identifies prevalent issues, such as limited standardization, difficulty in updating ontologies to reflect the latest medical insights, and obstacles in integrating heterogeneous datasets. Additionally, gaps are noted in addressing patient privacy and ethical concerns, which are crucial in healthcare applications. This review contributes by highlighting these challenges and proposing areas for further research, such as developing adaptable, scalable ontologies that are ethically aligned and capable of supporting advanced technologies like AI. The findings underscore the need for collaborative efforts among healthcare providers, data scientists, and policymakers to build robust ontology frameworks that can sustainably support healthcare advancements.

## 1 Introduction

A formal, structured presentation of data in a particular field that includes a collection of ideas, their descriptions, and the connections between them is called an ontology. It provides a universal language and framework for comprehending and learning about the domain, acting as a blueprint for modelling the things and their relationships. To make data sharing, retrieval, and interoperability between systems easier, ontologies are frequently utilized in domains such as artificial intelligence, data integration, and semantic web technologies. Ontologies allow both humans and machines to handle and analyse complicated data in an organised and meaningful fashion by clearly defining the sorts of entities, their characteristics, and their interactions.

*Ontology Set Representation*: O = (C, R, I, H).

C is a set of concepts (or classes), R is a set of relationships between concepts, I is a set of instances (or individuals, examples of concepts), H is a set of hierarchical relations that organize concepts in a tree-like structure.

*Hierarchical Relationship (Taxonomy)*: ∀c_i_, c_j_∈C, (c_i_ ≺ c_j_) ⇒ c_i_ is a subclass of c_j_, where c_i_ ≺ c_j_ denotes that c_i_ is a **.

**Figure 1:**
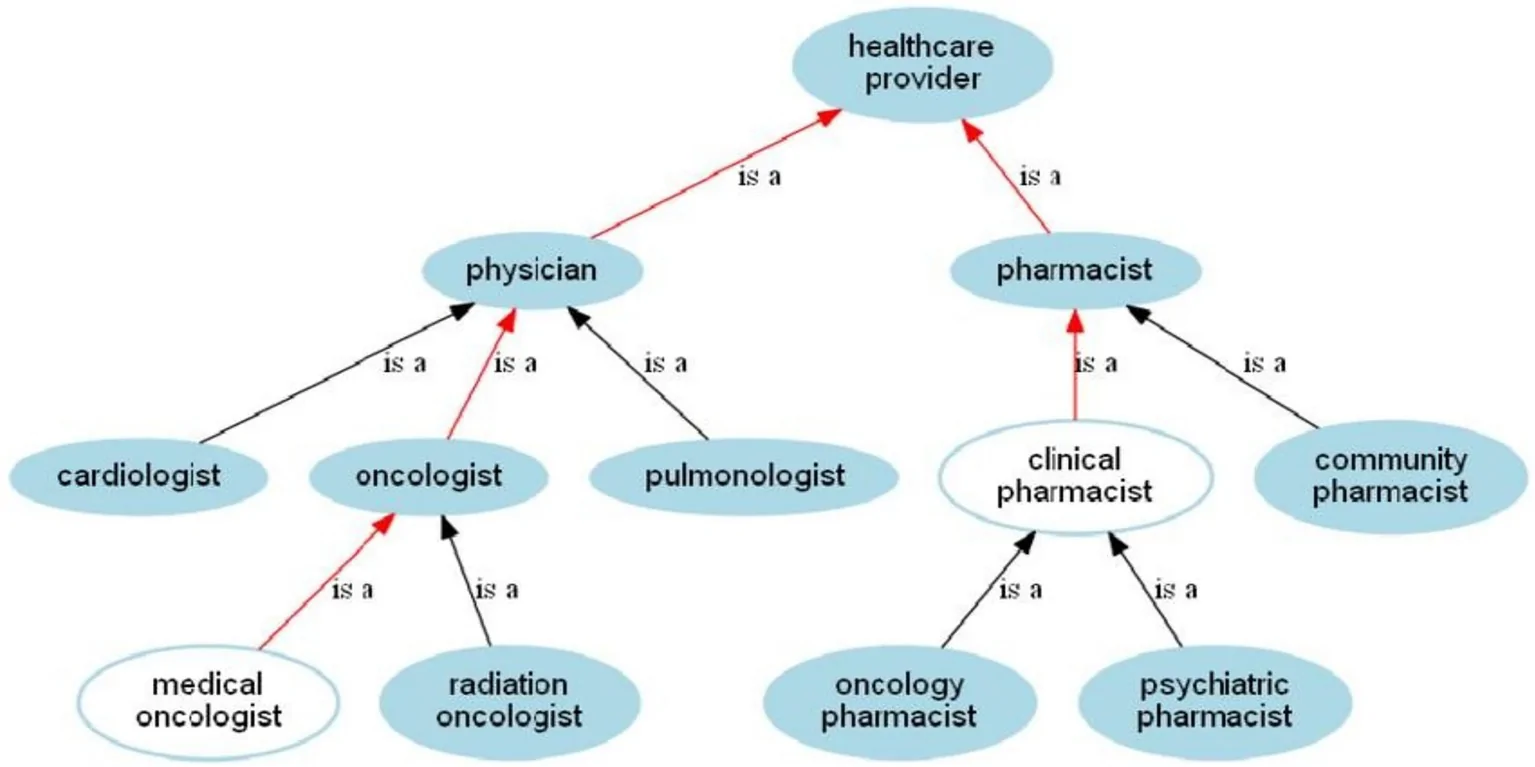
Structure of medical ontology.

Healthcare is the systematic delivery of medical services to people or communities, including a variety of services meant to maintain, improve, or promote health. Provided by medical professionals, including physicians, nurses, pharmacists, and therapists in a variety of settings, including clinics, hospitals, and community health centres, it covers preventative, diagnostic, therapeutic, rehabilitative, and palliative care services. Enhancing quality of life, managing illnesses, and guaranteeing access to essential medical resources are the goals of healthcare, which frequently operates within a framework of laws, technological advancements, and public health campaigns that promote population longevity and well-being. In the healthcare industry, ontology is a systematic framework for managing, organizing, and representing medical knowledge. It facilitates improved data retrieval, analysis, and integration across various healthcare systems. It offers a methodical approach to defining and classifying healthcare concepts, including illnesses, symptoms, medical procedures, treatments, healthcare professionals, and facilities, as well as the connections among them. Ontologies can be used to standardize and link healthcare data, which facilitates consistent interpretation of complicated medical information by researchers, healthcare practitioners, and computing systems. By providing a shared knowledge of medical terminology and relationships, ontologies in the healthcare industry promote interoperability and enable successful communication between various healthcare information systems, as mentioned in Figure 1.

In order to create a thorough and easily navigable knowledge base, an ontology might, for instance, describe a concept such as “hypertension” together with its associated symptoms, causes, risks, and potential therapies. Effective data mining, along with knowledge finding applications, is made possible by this relational and hierarchical structure, which supports research, clinical decision-making, individualized care, and public health campaigns. In addition, ontologies are essential to electronic health records (EHRs) because they guarantee that patient data is processed, saved, and retrieved in a consistent manner, enhancing data quality and making it possible to automate clinical guidelines, warnings, and reminders. Leading examples of healthcare ontologies are the Gene Ontology (GO), which concentrates on molecular activities, biological processes, and cellular components, and the SNOMED CT (Systematized Nomenclature of Medicine–Clinical Terms), which standardizes clinical terminology. In a rapidly evolving digital and data-driven world, healthcare ontologies are a fundamental tool for improving patient outcomes, improving medical studies, and streamlining healthcare delivery by creating a structured knowledge model that can change in tandem with new scientific findings and medical breakthroughs.

## 2 Background study

**Figure 2:**
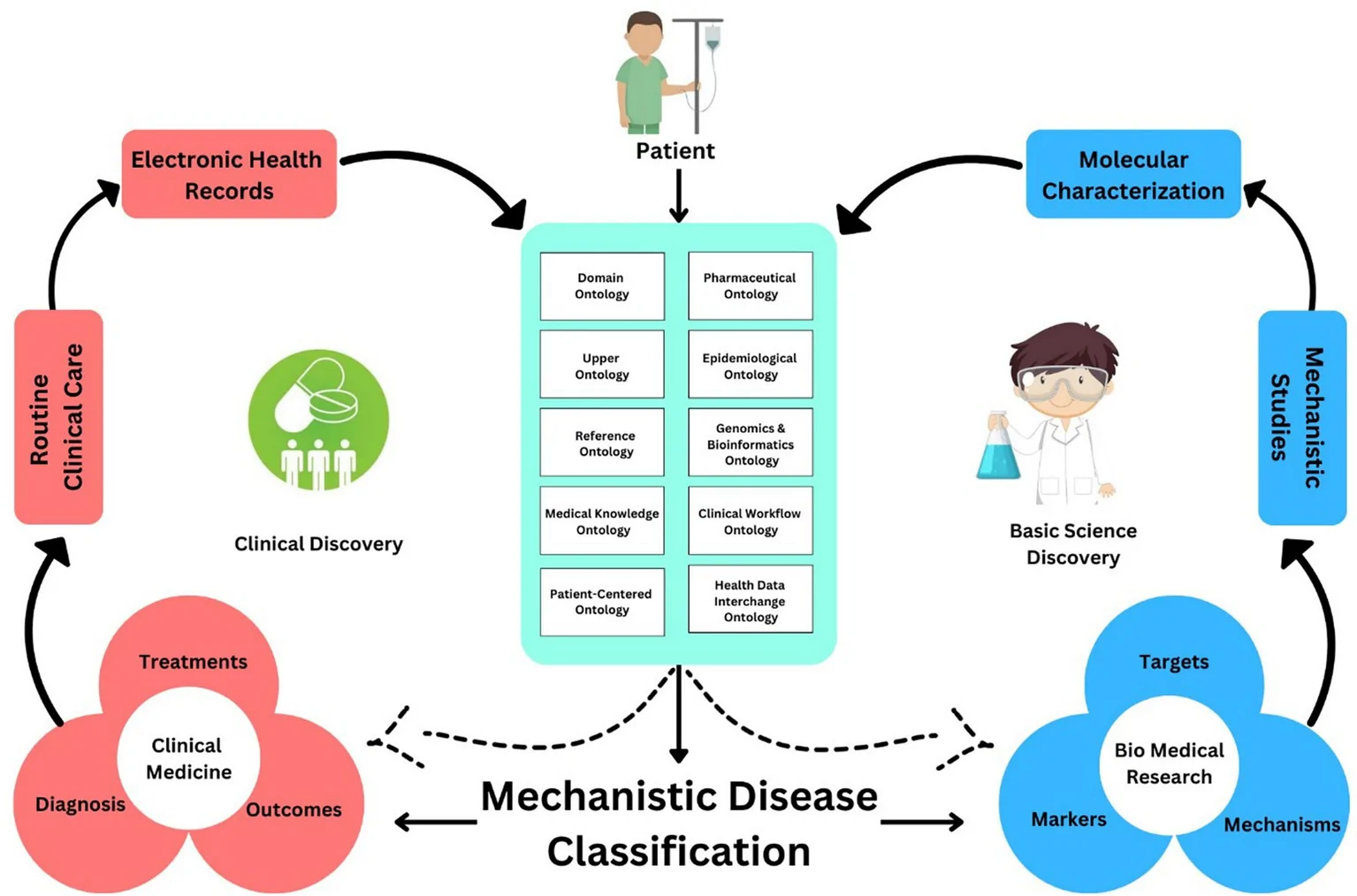
Classification of medical ontology in healthcare.

The background study provides an in-depth exploration of the various approaches and strategies applied in healthcare through ontology. It focuses on using ontologies to enhance diagnosis, treatment, and patient care by organizing medical knowledge systematically. By defining and linking healthcare concepts such as diseases, symptoms, and treatments. Ontologies support effective data integration, interoperability, and decision-making, facilitating improved clinical outcomes and a more comprehensive, standardized approach to patient care and medical research, as mentioned in Figure 2.

### 2.1 Systematic review methodology

This review was conducted following the Preferred Reporting Items for Systematic Reviews and Meta-Analyses (PRISMA) guidelines to ensure a transparent and reproducible review process. A comprehensive literature search was performed using major scientific databases, including Scopus, Web of Science, PubMed, IEEE Xplore, ScienceDirect, SpringerLink, ACM Digital Library, and Google Scholar. The search combined keywords such as heart disease prediction, medical ontology, artificial intelligence, machine learning, deep learning, clinical decision support, electronic health records, and knowledge graph. Studies published in English between 2015 and 2025 were considered. Duplicate records were removed before screening. Titles, abstracts, and full texts were independently evaluated according to predefined inclusion and exclusion criteria. Only peer-reviewed articles focusing on ontology-based artificial intelligence methods for heart disease prediction were included in the final review.

#### 2.1.1 Domain ontologies

Domain ontologies capture knowledge specific to a particular domain of healthcare, such as diseases, treatments, or anatomy. They provide a structured vocabulary for the concepts and relationships within that domain.

C={x∣xisadomain−specific concept}

#### 2.1.2 Upper ontologies

Upper ontologies provide general concepts and relations that apply across various domains. They help standardize the structure of ontologies and ensure compatibility and interoperability between different systems.

∀x(Entity(x)→x∈E)

#### 2.1.3 Reference ontologies

Reference ontologies provide a foundation for creating domain-specific ontologies by establishing standard terms and concepts across different areas of healthcare. They are often adopted as standards.

C={Disease,Symptom,Patient,Diagnosis,Treatment}

#### 2.1.4 Medical knowledge ontologies

These ontologies focus on representing medical knowledge, such as clinical guidelines, diagnostic criteria, and decision-making pathways. They provide valuable resources for medical professionals and researchers.

$$C=\{\begin{array}{l} \text{Disease},\text{Symptom},\text{Patient},\text{Diagnosis},\\ \text{Treatment},\text{Medication},\text{Anatomical Part} \end{array}\}$$

#### 2.1.5 Patient-centred ontologies

These ontologies represent data about patients, including their demographics, medical history, and clinical data. They help in personalizing care, managing patient data, and ensuring better patient outcomes.

$$C=\{\begin{array}{l} \text{Patient},\text{Health Condition},\text{Symptom},\\ \text{Treatment Plan},\text{Medication},\text{Health Goal},\\ \text{CareTeam Member},\text{Medical History Record} \end{array}\}$$

#### 2.1.6 Pharmaceutical ontologies

These ontologies focus on drug-related knowledge, such as pharmaceutical substances, formulations, side effects, and interactions. They are often used in pharmacology, drug development, and personalized medicine.

$$C=\{\begin{array}{l} \text{Drug},\text{Active Ingredient},\text{Dose Form},\text{Disease Target},\\ \text{Side Effect},\text{Drug Interaction},\text{Patient},\text{Therapeutic}\;\mathrm{Use} \end{array}\}$$

#### 2.1.7 Epidemiological ontologies

These ontologies represent knowledge related to the spread and control of diseases in populations, including risk factors, patterns of disease transmission, and preventive measures.

P={x∣xbelongs to the study population}

#### 2.1.8 Genomics and bioinformatics ontologies

These ontologies are specifically designed to manage and represent genomic and biological data, including genes, proteins, diseases, and their interrelationships.

G={x∣Gene(x)}

#### 2.1.9 Clinical workflow ontologies

These ontologies represent the flow of processes within clinical settings, including patient care pathways, diagnostic steps, and treatment plans. They are crucial for supporting clinical decision support systems (CDSS) and healthcare process automation.

CP={x∣ClinicalProcess(x)}

1. Health Data Interchange Ontologies

These ontologies support interoperability and data exchange between different healthcare systems. They provide standardized definitions and formats for exchanging health information.

HR={h∣HealthRecord(h)}

## 3 Literature review

Reviewing 20 works on healthcare ontologies from 2009 to 2018, it highlights tools including Protege, SPARQL, and SNOMED CT, and categorises methodologies, systems, and ontology extensions. The paper outlines research gaps for further development and emphasizes the significance of ontology in health services (Okikiola et al., 2020). Reviewing the mobile client, smart home server, and web-based context generator, all integrated into the clever home healthcare system prototype. The reasoning engine processes the home environment contexts that the context generator generates to initiate functions, and the mobile client displays the outcomes for control (Lee and Kwon, 2013). The article introduces a new ontology that was created through conceptual modelling and a survey of the literature to categorize information disruption attacks in IoT healthcare systems. The ontology, which is implemented in Protege 5.5.0, is assessed for accuracy and consistency with the goal of improving comprehension and field response to such attacks (Jamshed, 2021). The article proposes a framework using smartphones to integrate medical sensors, addressing interoperability challenges with ontologies and IEEE 11073 standards to enhance healthcare and data integration, particularly for the elderly and underserved populations (Hennessy et al., 2013). The article introduces a healthcare monitoring system using ontology agents in the JADE platform, integrating wireless sensors for real-time alerts and data sharing to enhance proactive healthcare and community care (Christopoulou et al., 2016). The article proposes a three-stage data acquisition and annotation process, using a hierarchical ontology to improve labelling accuracy and enhance activity recognition for healthcare applications in home settings (Woznowski et al., 2016).

The article presents a Multi-Agent Information System (MAIS) in Hong Kong, using ontology and a patient-centred approach to autonomously manage tasks and improve healthcare coordination and service quality (Sun et al., 2011). The article introduces VnHIES, an ontology-based system that enhances semantic extraction from healthcare web documents, addressing challenges in entity categorization and disease name extraction, while handling language-specific issues in Vietnamese text processing (Dung and Kameyama, 2007). The article introduces a method for improving healthcare interoperability by transforming data into the HL7 FHIR standard through ontology matching, utilizing syntactic and semantic similarity measures to enhance accuracy and integration (Kiourtis et al., 2019). The article introduces an ontology-based security framework for healthcare web applications, focusing on early identification of vulnerabilities and integrating specialized protocols to enhance security in the healthcare sector (Alenezi, 2021). The article introduces an ontology-based e-learning framework for healthcare human resource management, using Semantic Web technologies (Bajenaru et al., 2016). The article introduces an ontology-based approach to enhance patient privacy and compliance in decentralized healthcare systems using IoT, proposing a HealthCare Security and Privacy (HCSP) ontology to monitor compliance and address privacy risks (Kanaan et al., 2017). The article presents an ontology-based knowledge management framework to enhance health information systems (HIS) by capturing tacit knowledge, improving information flow, and optimizing patient care and communication in clinical settings (Fareedi and Ghazawneh, 2018).

The document introduces an ontology-based e-learning system for healthcare human resource management (HHRM) in Romania, using semantic web technologies to provide personalized training for managers and improve decision-making and public health outcomes (Bajenaru and Smeureanu, 2015). The article explores the integration of ontology and deep learning in healthcare systems through the Cardiovascular Disease Ontology (CVDO) to improve early detection and personalized management of cardiovascular diseases (Divakar et al., 2019). The article presents an ontology-driven multi-agent approach to enhance interoperability in e-Health systems, improving information sharing and clinical decision-making across electronic health record (EHR) systems (Ying et al., 2010). The article introduces a patient-centric interoperability framework using ontology to enable secure access to health information, improving patient engagement, and facilitating data integration across healthcare providers (Azarm and Peyton, 2018). The article introduces the Ontology for Simulation Modelling of Population Health (SimPHO), a framework designed to enhance the clarity, interoperability, and reproducibility of population health simulation models in research and policy-making (Okhmatovskaia et al., 2012). The Article introduces an ontology to improve clinical data quality in telemedicine, proposing a framework to adapt treatment protocols based on data quality issues to ensure safe patient care (Larburu et al., 2015). The article presents a framework utilizing domain ontologies and quality metrics for automated data quality assessment in healthcare, enhancing decision-making by detecting anomalies and ensuring data reliability (Urrutia et al., 2017). This article presents an ontology-based IoT healthcare system for elderly patients with chronic diseases, integrating clinical guidelines and ontologies like SNOMED CT and ICD-10 (Sondes et al., 2019). It enables real-time monitoring, emergency detection, and accurate health assessments through data integration, semantic interoperability, and rule-based reasoning. This article describes an ontology-based system for personalized care in chronic illness management, adapting clinical data and treatment plans to individual patient needs (Sondes et al., 2019). Validated by the K4CARE project, the system improves decision-making and care coordination, gaining high satisfaction from healthcare professionals. The article details an ontology for identity resolution in healthcare, designed to improve data interoperability and record linkage by addressing semantic and syntactic heterogeneity across systems (Duncan et al., 2015). Integrating models like SEM and CEM, it facilitates the accurate management of identity data over time.

The article critiques the limitations of the biomedical model, advocating for a holistic, humanized approach that integrates sociological, cultural, and emotional aspects of care (Contatore et al., 2017). It emphasizes the importance of multiprofessional collaboration and patient-centred, reflective practices in modern healthcare. The article discusses the application of relational ontology in preventive healthcare, introducing a Hierarchy of Attributes and Concepts (HAC) model to improve clustering and data organization (Suh and Komatireddy, 2011). This approach enhances inference capabilities and uncovers new relational knowledge, contributing to more effective healthcare solutions. The article explores collaborative ontology engineering to develop healthcare decision support systems, using case studies to demonstrate how structured, reusable knowledge enhances decision-making through stakeholder-driven, iterative processes (Spoladore and Pessot, 2021). The article compares Allegro Graph and Oracle 12c for healthcare data management, evaluating their performance with healthcare ontologies to identify the best triple store for semantic datasets (Can et al., 2017). The research aims to enhance data accessibility and integration in healthcare applications. The article presents Compo PHC, an ontology-based semantic web system aimed at improving access to medical knowledge for healthcare professionals in Brazil’s primary care sector (Moraes et al., 2012). It highlights the transition from paper-based systems to web tools to enhance knowledge retrieval, patient care, and operational efficiency. The article introduces a semantic web ontology-based approach for dynamic healthcare service composition, utilizing contextual information to improve service selection (Subbulakshmi et al., 2019). The framework aims to enhance patient outcomes by providing quick access to relevant medical services during emergencies. The article emphasizes the role of ontology-based approaches in health information systems, focusing on knowledge organization and representation (Meraji et al., 2013). It also addresses challenges in creating accurate healthcare ontologies and their integration in system design and evaluation to improve decision-making and service provision. The article proposes an ontology-based E-Healthcare Decision Support System to enhance healthcare services in India by improving data analysis, patient care, and decision-making (Vyas and Pal, 2012). It also addresses challenges like computer illiteracy and funding issues in rural areas, while emphasizing the benefits of reduced medical errors and increased patient involvement. The article explores the development of a centralized semantic knowledge base in India, integrating heterogeneous ontologies to improve healthcare data management and decision-making (Sunitha and Suresh Babu, 2014). It aims to enhance public health outcomes by supporting policymakers with a robust framework using RDF and OWL technologies. The article presents a bottom-up approach for ontology extraction in healthcare security, using fuzzy clustering to classify XML documents and enrich existing ontologies (Indumathi and Uma, 2008). It emphasizes the importance of risk management and the role of ontologies in defining security policies for adaptable healthcare systems.

The article introduces the Health Condition Evolution Ontology (HECON), using a Knowledge Graph to connect health conditions to SNOMED CT concepts, facilitating better decision-making in emergencies (Tirado et al., 2022). It employs machine learning techniques to extract data from Health Condition Statements, supporting intelligent systems in evaluating health records. The article focuses on creating a critical knowledge ontology for healthcare organizations to protect sensitive clinical data, emphasizing CIA-P principles and structured knowledge categorization (Pereira and Santos, 2013). It utilizes topic modeling and ontology engineering, with exploratory focus group research to define key concepts and relationships. The article presents a two-layer telemonitoring architecture utilizing ontologies for unified data representation and secure communication between home and healthcare sites, targeting improved care for chronic patients (Lasierra et al., 2010). It details conceptual and physical layers that enable flexible, secure data flow and demonstrates practical data integration within the system to support patient monitoring and care management. The article introduces an ontology-based provenance management system that combines the Open Provenance Model with Ontology-Based Access Control to improve healthcare data privacy (Can and Yilmazer, 2020). By tracking access histories and enforcing fine-grained policies, the system protects sensitive patient information, with a case study on HIV diagnosis demonstrating its effectiveness in upholding confidentiality and regulatory compliance. The article proposes an intelligent health diagnosis technique that improves diagnostic accuracy by using automatic ontology generation, web-based personal health records, and deep learning to analyze disease-symptom relationships and user lifestyle factors (Kim and Lee, 2019). This method outperforms traditional approaches, addressing the limitations of manual knowledge compilation and enhancing healthcare decision-making. The Context-aware Activity Manipulation Engine (CAME) leverages ontology and semantic reasoning to enhance ADL recognition and decision-making in Alzheimer’s care, integrating contextual data for improved accuracy and responsiveness (Khattak et al., 2011). The article analyzes the impact of climate change on food security, predicting reduced crop yields and food shortages, and emphasizing the need for agricultural adaptation strategies (Wang et al., 2020). The article proposes a knowledge-based artifact using lean principles to reduce inefficiencies in healthcare, particularly radiology, validated through real-world application, and suggests broader implementation for improved operational efficiency (Musa and Othman, 2016). The article examines integrating FHIR standards with distributed ledger technology to enhance semantic interoperability and secure data sharing in healthcare (Li et al., 2019). It proposes a smart contract framework using FHIR terminologies to enable structured data transactions, aiding machine learning-based anomaly detection for improved data security. The article presents a Multi-Agent System (MAS) architecture to improve EHR security, privacy, and interoperability by standardizing data integration through ontologies and managing access with security tags (Wimalasiri et al., 2004). This approach leverages network security technologies to facilitate secure information exchange, enhancing patient care quality. The article by Zeshan and Mohamad examines using medical ontologies with service-oriented architecture to improve interoperability and information sharing in healthcare, focusing on entity extraction and taxonomy creation for structured communication (Zeshan and Mohamad, 2012). Challenges include coordinating with domain experts, with future plans for stakeholder feedback to assess scalability and effectiveness. The article presents a mobile Cyber Physical System (CPS) for real-time health monitoring of elderly individuals, utilizing wearable devices and embedded systems for remote tracking and emergency communication (Costanzo et al., 2016). It incorporates a user model ontology for interoperability and fuzzy rule-based diagnostics to enhance decision-making in health emergencies.

The article addresses the challenge of monitoring elderly individuals’ health at home, noting gaps in existing systems that lack real-time tracking and remote communication (Parvan, 2016). It advocates for a comprehensive solution integrating wearable devices, environmental sensors, and intelligent algorithms to enable timely interventions. The article presents the MIPO framework, which combines knowledge graphs and Transformer-based architecture with medical ontologies to improve predictive tasks in healthcare (Peng et al., 2021). It addresses challenges in NLP applications, such as limited unlabelled data and model interpretability, aiming to enhance decision-making and patient outcomes. The article uses an ontological framework to examine India’s healthcare policies, revealing strengths in information and accessibility but gaps in technology adoption and insurance coverage (Sastry et al., 2017). It suggests the need for a more systematic approach to improve policy implementation and healthcare delivery. The article proposes an Audit Rule Ontology for Healthcare Decision Support Systems, combining Continuous Auditing with a Healthcare Common Ontology to enhance process definition, interoperability, and automated auditing across diverse healthcare data sources (Subhani and Kent, 2014). The article proposes an ontological framework to standardize and digitize Clinical Pathways in Health Information Systems, improving interoperability, communication, and healthcare delivery (Alahmar et al., 2020). The article presents an ontological framework for managing non-functional requirements and device characteristics in remote healthcare systems, aiming to improve patient care through better integration and monitoring (Koay et al., 2009). The article proposes an ontological framework for mapping healthcare research, focusing on Medical Use of Health Information Systems (MUHIS) (Ramaprasad and Syn, 2014). By categorizing 200 articles from PubMed, it identifies “bright,” “light,” and “blind/blank” spots in healthcare knowledge, highlighting research gaps and future opportunities. The article discusses an intelligent U-healthcare system that integrates wearable devices, mobile systems, and Clinical Decision Support Systems (CDSS) for monitoring and managing health data (Ko et al., 2007). It highlights three service scenarios—Diagnosis, Remote Monitoring, and Emergency Management—demonstrating enhanced healthcare accessibility and quality.

The article reviews multi-agent systems in healthcare, focusing on human and software agents, and the use of ontology and terminology servers to enhance interoperability and semantic coherence (Falasconi et al., 1997). It observes that while terminology servers are well-developed, ontological services are still emerging, but are essential for effective clinical data management. The article introduces an ontology-based context information model for u-healthcare services, classifying healthcare data into seven categories to address scalability and personalization issues (Ryu et al., 2011). The model integrates diverse context data and supports personalized healthcare, demonstrated using the OSGi framework for high-level context inference. The article introduces an Ontology-Based Access Control (OBAC) model for securing cloud-based Personal Health Records (PHR), using SNOMEDCT and vCard ontologies for structured, decentralized access control with centralized management. Attribute-Based Encryption (ABE) enhances security, and future plans include inference engines to refine authorization (Mohan and Aramudhan, 2015). This article explores the role of Ontology, particularly SNOMED CT, in enhancing Electronic Medical Records for improved decision-making, addressing challenges in Ontology construction and information extraction (Julina and Thenmozhi, 2012). It proposes a structured approach using Bayesian Belief Networks to support decision-making and emphasizes the need for semantic interoperability to improve clinical outcomes.

The article proposes an ontology-based IoT healthcare system to enhance emergency care by integrating and interpreting data across hospital systems, facilitating real-time decision-making and access to patient and resource information (Kumar, 2015). This system aims to improve diagnostic speed and treatment accuracy in critical situations. The article proposes an ontology-based model to improve semantic interoperability in IoT healthcare systems, facilitating seamless data exchange across diverse systems (Ahamed and Chishti, 2021). Their approach aims to address data heterogeneity and enhance decision-making to support accurate diagnosis and treatment. The article proposes an ontology-based model for EHR interoperability, aiming to standardize data sharing across healthcare providers and improve public health outcomes through structured data integration (Rao et al., 2014). While effective for richer search results, it notes limitations in external ontology connectivity, which may isolate the knowledge base.

The article presents a semantic middleware architecture to integrate IoT data with EHR systems, addressing challenges in data sharing and enhancing real-time patient monitoring and care. It highlights the potential to improve patient outcomes, reduce healthcare costs, and enable proactive medical interventions (Alamri, 2018). The article introduces A-SHIP, an ontology-based framework for adaptive healthcare insurance that customizes policies based on individual health needs and integrates IoT and cloud computing for efficient data management (Al-Thawadi et al., 2022). It emphasizes a customer-centric approach to improve service delivery, patient outcomes, and cost optimization. The article presents an ontology-based competence management system to improve training efficiency and human resource management in healthcare by systematically assessing competencies and aligning training with strategic planning (Kunzmann and Schmidt, 2006). The article introduces an Ontology-Based Context-Aware Service Engine for U-health applications, using ontology to enhance knowledge sharing, semantic interoperability, and decision-making for contextually relevant health services (Ko et al., 2006). The article discusses an ontology-based ehealth system using Semantic Web technologies to recommend personalized Thai herbs based on user health data, symptoms, and preferences (Kato et al., 2009). The article explores using a BILSTM-CRF model for healthcare named entity recognition (HNER) from Twitter, enhancing accuracy with preprocessing, UMLS tagging, and word embeddings for public health research (Batbaatar and Ryu, 2019). The highlight advancements in software engineering, focusing on quality assurance, agile methodologies, project management, and the integration of new technologies to improve software development practices (Kuziemsky et al., 2003). Ontology-based framework that enhances COPD management by integrating real-time monitoring, semantic data, and decision support for improved patient outcomes (Ajami and Mcheick, 2018). Introduced a method to structure unstructured patient data from online forums, addressing challenges of informal language in health discussions. It emphasizes the use of visualization tools to improve data accessibility for clinical research and decision-making (Sampathkumar et al., 2015). Ontology-based tool designed to improve knowledge management in Brazil’s PHC system, found useful by 80% of users despite connectivity and complexity challenges (Moraes et al., 2012). The study emphasizes formal knowledge representation to support better healthcare decision-making and outcomes. Developed a personalized healthcare model for cancer patients using ontology alignment, integrating medical, economic, and social factors to enhance decision-making (Chakrabarty and Roy, 2016). The approach, utilizing OWL and alignment algorithms, demonstrates improved precision and recall in treatment recommendations, with potential for optimizing chronic illness management. The article presents an AI planning-based approach to healthcare dialogue management, integrating a conversational ontology to automate real-time, information-seeking dialogues (Teixeira and Dragoni, 2023). It evaluates scalability and real-time applicability, noting potential limitations that could be addressed with future optimization strategies. The article introduces the HIS-RA Ontology to improve Software Product Lines (SPL) in Healthcare Information Systems (HIS) by addressing non-functional requirements and capturing variability (Losavio and Ordaz, 2016). It supports the discovery of web services and semi-automatic generation of architectural configurations, aiding complexity management in HIS development.

It examines semantic heterogeneity in healthcare, highlighting issues like inconsistent reports and incompatible terminology systems that hinder interoperability (Ganiyat et al., 2013). It proposes an ontology matching framework to enhance semantic interoperability and improve communication and data exchange across healthcare systems. S-Trans is a method for converting XML-based healthcare data (XSD/DTD) into OWL ontologies, improving semantic interoperability by addressing duplicate elements (Thuy et al., 2012). It uses a novel similarity measure based on relationships and cardinality to decide whether to merge or distinguish concepts, enhancing ontology accuracy and completeness. Ontology-based SOA framework to enhance interoperability in TCM healthcare by connecting legacy systems through layers for service discovery, operations, data synchronization, and security (Chen et al., 2012). This framework aims to standardize TCM practices, improve information management, and address terminological inconsistencies in the field. This study applies Enterprise Ontology (EO) and the Design Engineering Methodology for Organizations (DEMO) to identify and redesign inefficient healthcare transactions, particularly in Emergency Departments and Pharmacies, enhancing inter-organizational collaboration and service quality (Dias et al., 2012).

The Context-embedded Intelligent Hospital Ontology (CIHO) using OWLDL to enhance context awareness and interoperability in healthcare systems, addressing challenges of fragmented communication (Yao et al., 2009). By integrating RFID and sensor data, CIHO aims to improve real-time decision-making, patient safety, and care quality. This study introduces an ontology-based knowledge network for healthcare user training, combining ontology constructs with multimedia to promote process-oriented thinking (Macris et al., 2009). The approach aims to enhance critical thinking, knowledge dissemination, and user engagement in healthcare process modeling.

An ontology-based approach to healthcare website design, highlighting its benefits over traditional text-based sites in enhancing information retrieval and usability (Chang et al., 2022). The methodology, which involves structuring health resources with ontologies, aims to improve health communication and empower patients in managing their health. The Patient on IoT Framework integrates IoT and ontology-based models to enhance patient monitoring and decision-making, addressing issues like context-awareness and service quality (Zeshan et al., 2023). With an accuracy of 89.81%, it improves patient care, particularly for the elderly, by providing timely, data-driven medical recommendations. The article highlights the shift toward personalized healthcare, stressing the need for interoperable health information systems powered by ontologies to improve communication and integration (Blobel, 2011). It advocates for a framework that empowers patients to manage their health effectively within a collaborative care model. The article presents an intelligent system for constructing healthcare knowledge graphs, addressing challenges in integrating unstructured medical data with disease ontologies (Maghawry et al., 2022). By utilizing machine learning and NLP, the system aims to support diagnosis and disease prediction, enhancing healthcare delivery and patient awareness. The article reviews the use of ontology-based cybersecurity in health, emphasizing the need for dual ontologies to manage and protect sensitive health data (Belani et al., 2023). It advocates for extending existing ontologies and adhering to standards to enhance cybersecurity in complex healthcare environments.

**Figure 3:**
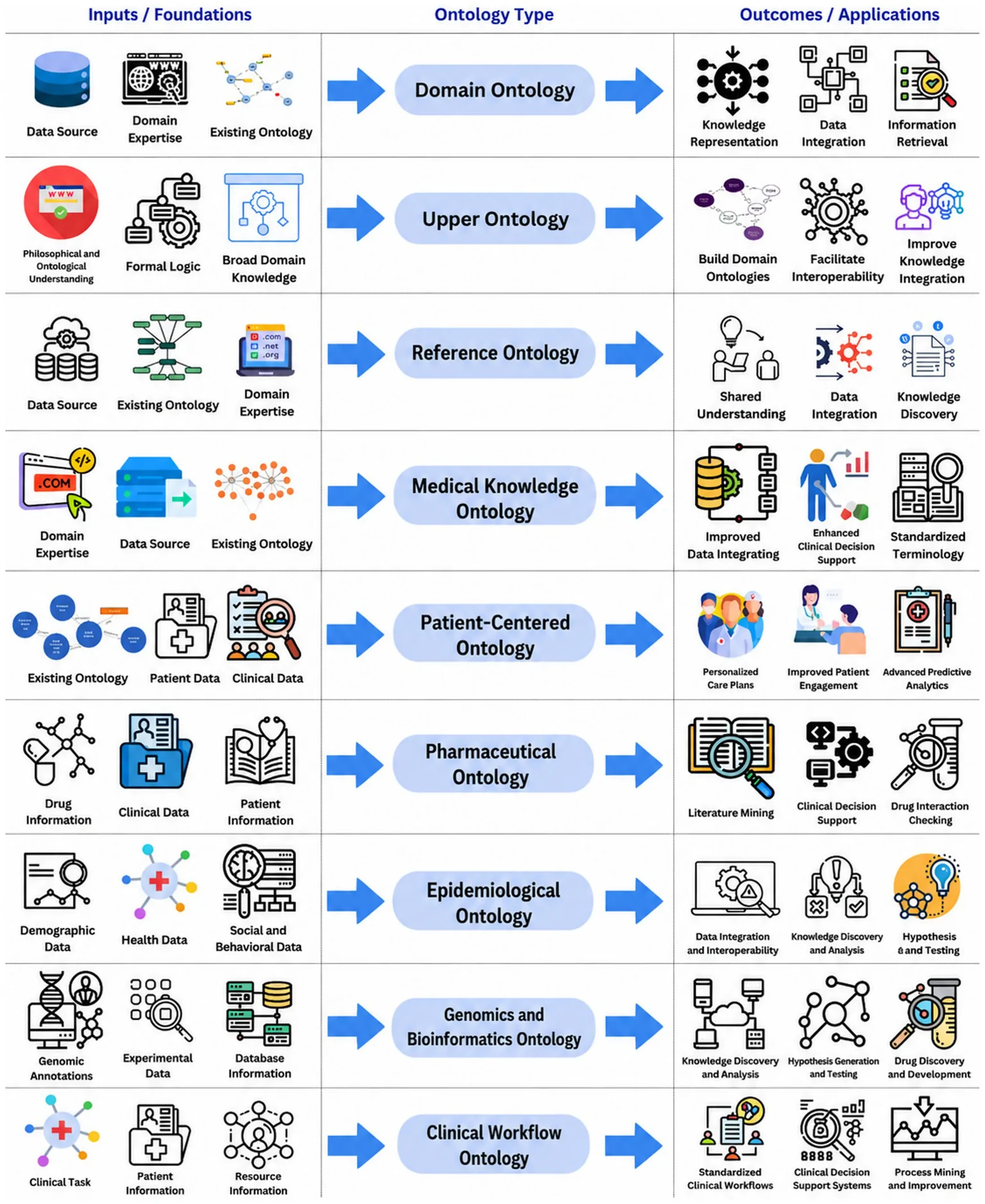
Architectural views of medical ontology in healthcare.

Ontology-based approach for requirements engineering in WBAH, focusing on extending SAREF4EHAW to address challenges like cybersecurity and device reliability (Belani et al., 2022). It highlights the need for a multidisciplinary approach to enhance IoT solutions supporting health and wellbeing, particularly during the COVID-19 pandemic. The article presents a schema ontology model to improve semantic interoperability in healthcare, addressing data heterogeneity for managing depressive disorder (DD) (Chong and Ali, 2021). It uses deep learning, semantic annotation, and microservices to enable seamless data integration for applications like remote monitoring and clinical decision support. Integrating patient data from web sources and hospital systems for healthcare decision-making, particularly in predicting cardiovascular health (Hole et al., 2023). It proposes an agent-based system using ontologies and Semantic Web technologies to improve early detection and healthcare outcomes. It explores the use of ontology in enhancing communication between humans and robots within the semantic web, focusing on RDF and URIs for data representation (Abhilash and Mahesh, 2022). It highlights the potential of ontology-based methods in healthcare for improving data organization and interoperability. The article critiques outdated humanistic definitions in health sciences, advocating for a redefined, nonbinary view of personhood that reflects contemporary social, technological, and ecological complexities (Holmes et al., 2024). Drawing on Cronenberg’s themes, it suggests a fluid, interconnected identity model to enhance nursing and health science paradigms in the Anthropocene. The article applies Clustering Based on Relational Ontology (CBRO) to improve preventive health care by organizing data into meaningful clusters, highlighting the relationship between nutrition and health conditions like diabetes and obesity (Suh and Gaddam, 2011). It suggests that CBRO can guide better nutritional practices and preventive strategies, with potential for expansion into other domains. Recent advances in Artificial Intelligence (AI), Knowledge Graphs, HL7 FHIR, and Large Language Models (LLMs) have significantly expanded the scope of ontology-based healthcare research. These emerging technologies have improved semantic interoperability, clinical decision support, and intelligent healthcare applications. A summary of recent developments, their healthcare applications, key contributions, and remaining research gaps is presented in Table 1. The overall study defines the architectural process flow of Medical Ontology in Healthcare as mentioned in Figure 3. Also, Figures 2, 3 are intended to provide a conceptual overview of the ontology ecosystem and healthcare ontology classifications discussed in this review. These figures summarize the relationships among key concepts, facilitate understanding of the reviewed literature, and provide a visual framework for interpreting subsequent discussions on ontology applications, interoperability, artificial intelligence integration, and future research directions.

**Table 1:** Recent advances in AI-enabled healthcare ontology research (2023–2025).

Reference	Technology/focus	Healthcare application	Key contribution	Research gap
Abu-Salih et al. (2023)	Healthcare Knowledge Graphs	Knowledge integration, semantic reasoning	Reviewed healthcare knowledge graph construction, applications, and future opportunities.	Scalability and real-time maintenance remain challenging.
Al Khatib et al. (2024)	Patient-Centric Knowledge Graphs	Personalized healthcare, clinical decision support	Integrated ontology, FHIR, NLP, and Generative AI for patient-centric healthcare.	Limited real-world clinical validation.
Tabari et al. (2024)	HL7 FHIR	Electronic Health Record interoperability	Reviewed FHIR-based semantic interoperability and health information exchange.	Integration with legacy systems remains difficult.
Msheik et al. (2024)	Knowledge Representation Models	Healthcare knowledge representation	Surveyed ontology and semantic web technologies for intelligent healthcare.	Standardization across heterogeneous healthcare systems remains limited.
Denecke et al. (2024)	Transformer Models / LLMs	Clinical NLP, medical decision support	Reviewed transformer and LLM applications in healthcare.	Explainability, privacy, and clinical reliability require further research.
Doneva et al. (2024)	Large Language Models	Biomedical text mining	Reviewed LLMs for biomedical text processing and knowledge extraction.	Hallucination, bias, and domain adaptation remain concerns.
Shaban et al. (2024)	LLMs + Knowledge Graphs	Knowledge graph construction	Reviewed integration of LLMs with knowledge graphs for semantic enrichment.	Reliable ontology alignment and factual consistency remain open challenges.

## 4 Methodology

A collection of rules known as the PRISMA model, or preferred report items for Systematic Studies and Meta-Analyses, is intended to enhance the Caliber and lucidity of research reviews and meta-analyses. It offers a precise, uniform method for summarizing the procedures and conclusions of these investigations. The PRISMA methodology aids readers and researchers in comprehending the selection, assessment, and integration of studies in the review by placing a strong emphasis on clear and consistent reporting.

### 4.1 Search process

**Figure 4:**
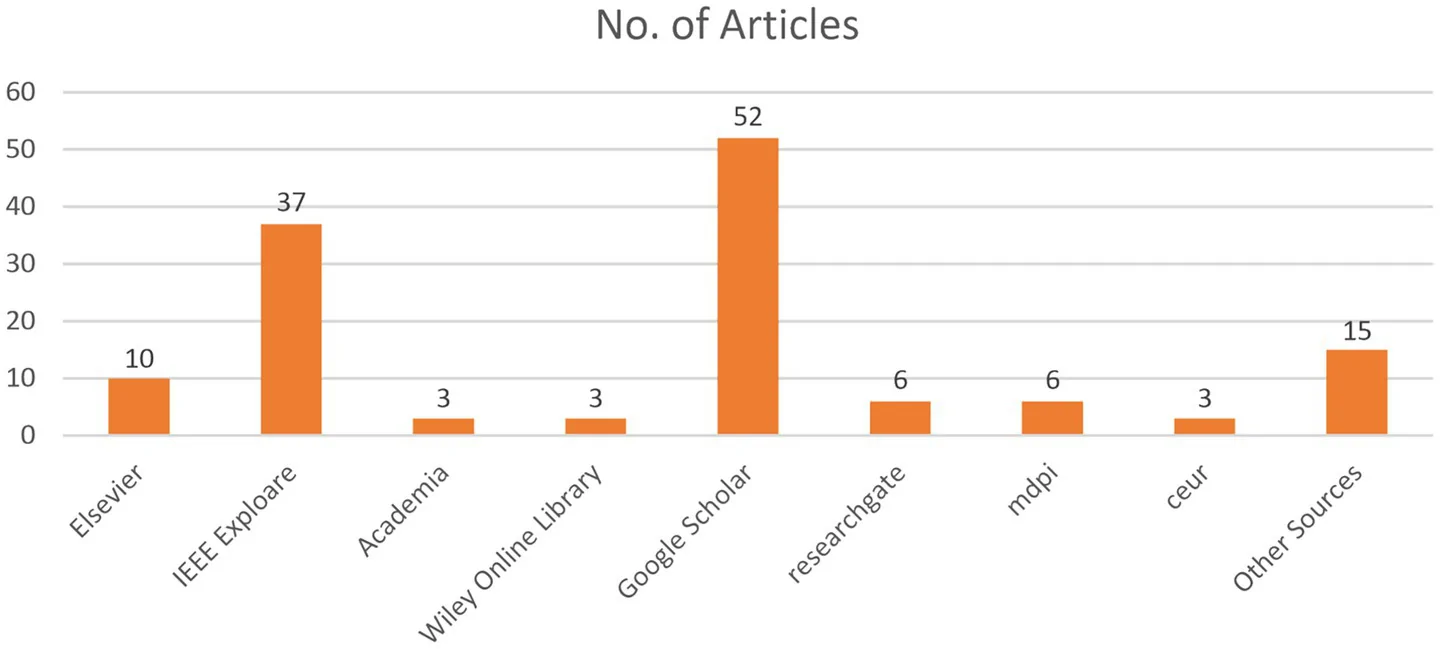
Graphical representation of list of research articles.

This review followed the Preferred Reporting Items for Systematic Reviews and Meta-Analyses (PRISMA) guidelines to ensure a transparent, systematic, and reproducible literature selection process. A comprehensive search was conducted across several reputable scientific databases, including Elsevier (ScienceDirect), IEEE Xplore, PubMed, Scopus, Web of Science, SpringerLink, Wiley Online Library, ACM Digital Library, MDPI, Google Scholar, ResearchGate, Academia, and CEUR Workshop Proceedings. These platforms were selected because they provide high-quality, peer-reviewed publications covering healthcare, artificial intelligence, semantic technologies, and ontology research. The distribution of articles collected from these sources is summarized in Table 2, while their graphical representation is shown in Figure 4. The search strategy combined healthcare ontology and artificial intelligence keywords using Boolean operators (AND, OR). Representative search terms included “healthcare ontology,” “medical ontology,” “clinical ontology,” “artificial intelligence,” “machine learning,” “deep learning,” “electronic health records (EHR),” “clinical decision support,” “semantic interoperability,” and “healthcare knowledge representation.”

**Table 2:** List of research articles searched in different databases.

Databases	Elsevier	IEEE Explore	Academia	Wiley Online Library	Google Scholar	ResearchGate	MDPI	CEUR	Other Sources
No. of articles	10	37	3	3	52	6	6	3	15

Studies were included if they: (i) focused on ontology applications in healthcare, (ii) addressed artificial intelligence, clinical decision support, semantic interoperability, or healthcare knowledge management, (iii) were published in peer-reviewed journals or conference proceedings, and (iv) were written in English. Studies were excluded if they were duplicate records, editorials, abstracts, non-English publications, or were not directly related to ontology-based healthcare applications. The study selection process involved five stages: identification, duplicate removal, title and abstract screening, full-text eligibility assessment, and final inclusion based on the predefined selection criteria. The complete workflow of the literature selection process is illustrated in the PRISMA flow diagram.

### 4.2 The criterion for including or excluding articles

The application of ontology in healthcare has been extensively studied. Certain criteria were established for selecting pertinent studies in order to maintain the review’s focus and manageability. These standards ensure that the literature evaluation is useful and well-targeted by limiting it to sources that most effectively support the primary objectives of healthcare ontologies. Criterion for Including: The research on ontology in healthcare and related tools or methods published between 2007 and 2024 was considered. When similar content appeared in multiple journals or conference proceedings, the most detailed version was selected for review. Criteria for Excluding: The selection procedure for ontology in healthcare excluded studies that did not fit the review’s focus or pertinent keywords, or those were published in unrecognized journals or conferences. The studies were identified, screened, and chosen for inclusion using a methodical process to guarantee that only the most reliable and pertinent sources were used.

### 4.3 PRISMA flowchart

**Figure 5:**
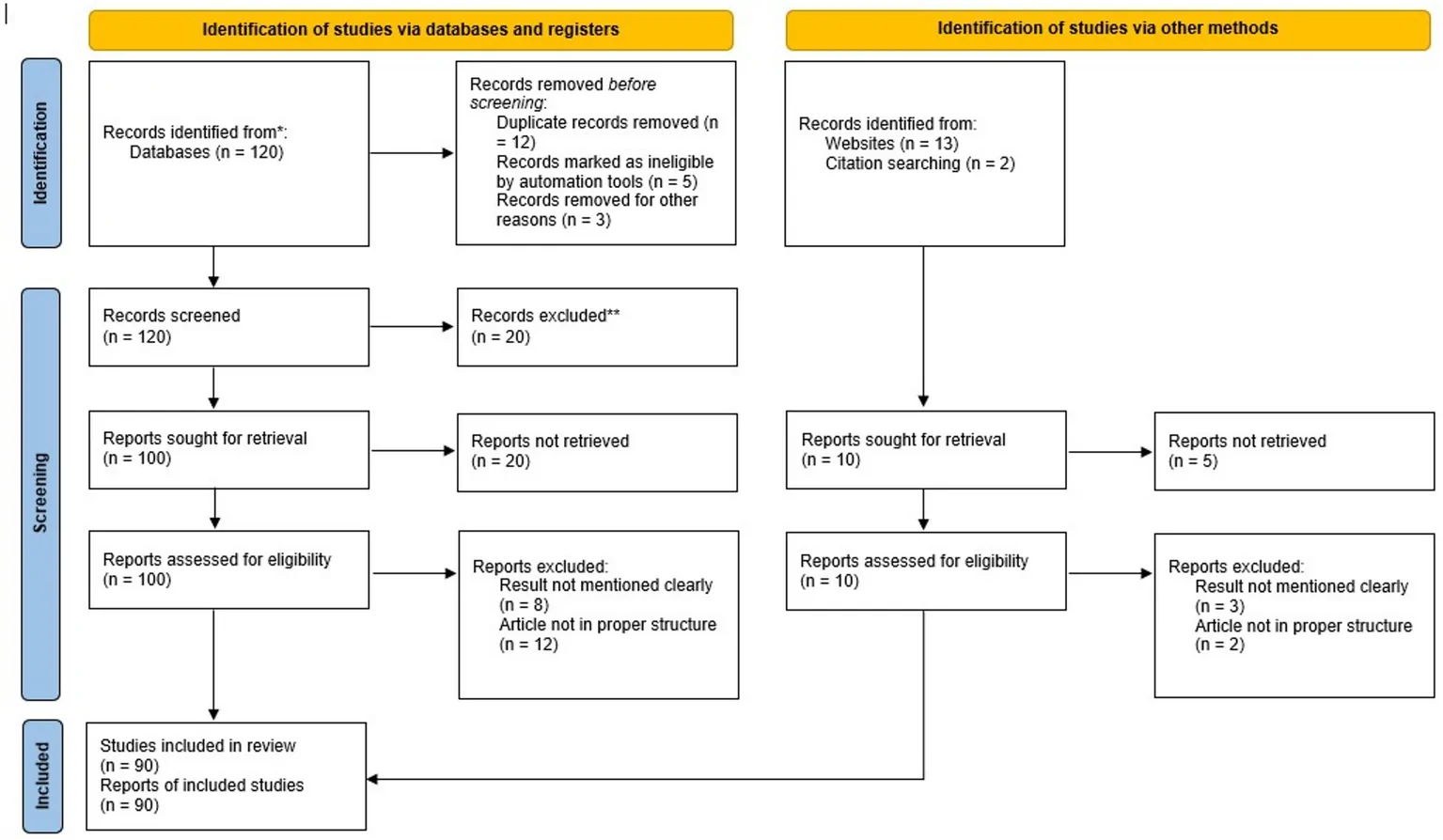
PRISMA flowchart for literature review.

The PRISMA (Preferred Reporting Items for Systematic Reviews and Meta-Analyses) flowchart, shown in Figure 5, visually represents the process of selecting studies for a systematic review or meta-analysis. It provides a clear and consistent way to track the number of studies found, checked, assessed for eligibility, and included in the final review. The flowchart has four steps—identification, screening, eligibility evaluation, and inclusion—which ensure that the selection process is thorough and can be repeated (Thangavel and Lourduswamy, 2021).

Table 3 summarizes the reviewed studies based on key aspects identified through the PRISMA-guided review process, including the research domain, objectives, dataset, methodology, reported performance, and identified challenges. The reported accuracy and performance metrics were extracted directly from the original publications as presented by the respective authors. Because the reviewed studies employed different datasets, methodologies, sample sizes, and evaluation protocols, these metrics were not statistically compared. Instead, they are presented to provide a qualitative overview of research trends and the reported effectiveness of ontology-based healthcare approaches.

**Table 3:** Shows the problems and difficulties from the literature survey.

S. No	Author name	Domain	Objectives of the research	Dataset	Methodology	Accuracy/result	Problem identified
1.	Hennessy et al. (2013)	HealthCare	Ontology for Patient Monitoring Integration.	Health Informatics	Framework Development Extraction and Synthesis.	89%	Cost, data, privacy, interoperability
2.	Christopoulou et al. (2016)	Healthcare	Ontology agent-based system for healthcare.	Sensors	Agent-Based Framework Architecture Design	74%	Data flow, collaboration.
3.	Dung and Kameyama (2007)	Healthcare	Accurate Healthcare Data with Ontology	Webpages	Data Collection, Pre-processing	97.03%	Disease extraction and NLP challenges.
4.	Kiourtis et al. (2019)	Healthcare	Ontology matching for HL7 FHIR interoperability	Triple store	Ontology, triple store, semantic similarity identifiers.	91%	Ontology matching errors, HL7 FHIR gaps.
5.	Mutambik et al. (2024)	Healthcare	Ontology for Healthcare Web Security.	Statistics	Ontology-based security management.	87%	Rising cyber threats to healthcare web apps
6.	Kanaan et al. (2017)	Ontology	HCSP Ontology for Patient Privacy and HIPAA.	Ontology	HCSP Ontology Development, Architecture.	35%	Privacy, security, and compliance in IoT healthcare
7.	Divakar et al. (2019)	Health	CVDO for prediction using deep learning.	Cardiovascular Disease Ontology.	Build, Implement, Test, Validate Ontology.	90%	Limited accuracy, delayed detection, and resource constraints.
8.	Azarm and Peyton (2018)	Healthcare	Ontology-Driven Patient-Centric Secure Framework.	Ontology	Scope, Reuse, List, Define, Entities.	78%	Fragmentation, Standardization, Healthcare Data Access.
9.	Larburu et al. (2015)	Telemedici ne	Ontology development for telemedicine safety.	Ontology	Elicit, Develop, Layer, Apply Ontology.	89%	QOD degradation in telemedicine settings.

### 4.4 Study quality consideration

A formal quality assessment or risk-of-bias evaluation was not performed because the objective of this study was to provide a comprehensive narrative review of ontology-based healthcare applications rather than a quantitative meta-analysis. Nevertheless, study quality was considered during the selection and interpretation process. Only peer-reviewed articles published in reputable journals and conference proceedings were included. Studies were evaluated based on their methodological clarity, relevance to healthcare ontology, implementation approach, experimental validation, and contribution to the research field. Furthermore, conclusions were drawn by considering the overall evidence across multiple studies rather than relying on the findings of individual publications.

The performance metrics summarized in Table 3 were extracted directly from the respective studies to provide an overview of the reported outcomes in ontology-based healthcare research. Since the reviewed studies employed different datasets, sample sizes, healthcare applications, methodologies, evaluation metrics, and validation protocols, the reported accuracy values should not be interpreted as direct quantitative comparisons. Instead, they provide a descriptive summary of the reported performance and help identify general research trends, strengths, and limitations across the existing literature.

To provide a broader perspective on the reviewed literature, Table 4 presents a thematic summary of ontology-based healthcare studies. The reviewed studies are categorized according to ontology type, healthcare application area, interoperability focus, security and privacy considerations, AI integration, implementation maturity, and future research directions. This thematic classification provides a clearer understanding of current research trends and highlights the remaining research gaps.

**Table 4:** Thematic summary of ontology-based healthcare studies.

Category	Summary of reviewed studies	Research trend/gap
Ontology type	Medical Ontology, SNOMED CT, ICD-10, HL7 FHIR, UMLS, Gene Ontology, Disease-specific Ontologies	Standard ontologies are widely used, but integration among multiple ontologies remains limited.
Healthcare application area	Disease Prediction, Clinical Decision Support, Electronic Health Records (EHR), Medical Diagnosis, Personalized Medicine, Healthcare Knowledge Management	Most studies focus on disease prediction, while fewer address comprehensive clinical decision support and personalized healthcare.
Interoperability focus	Semantic interoperability, EHR integration, data sharing across healthcare systems, and healthcare information exchange	Seamless integration across heterogeneous healthcare platforms remains challenging.
Security and privacy focus	Patient data protection, privacy preservation, secure healthcare data sharing, and access control	Comprehensive privacy-preserving ontology frameworks are still limited.
AI integration	Machine Learning, Deep Learning, Natural Language Processing, Knowledge Graphs, Explainable AI (XAI), Large Language Models (LLMs)	Integration of AI with ontology is increasing to improve prediction accuracy and explainability.
Implementation maturity	Conceptual Frameworks, Prototype Systems, Validated Clinical Implementations	Most studies remain conceptual or prototype; few have large-scale clinical validation.
Future research directions	Multimodal healthcare data integration, Explainable AI, Knowledge Graphs, Federated Learning, Clinical deployment	Future work should emphasize scalable, interoperable, explainable, and clinically validated systems.

### 4.5 Critical comparison of ontology-based healthcare applications

The reviewed studies demonstrate that ontology-based approaches have significantly improved semantic interoperability, knowledge representation, clinical decision support, and disease prediction in healthcare. Domain-specific ontologies provide an accurate representation of specialized medical knowledge, while upper and reference ontologies facilitate semantic consistency and interoperability across heterogeneous healthcare systems. Medical and patient-centred ontologies have been widely adopted to support clinical decision-making and personalized healthcare. Despite these advantages, several limitations remain. Many studies focus on conceptual frameworks or prototype implementations with limited validation using real-world clinical datasets. Interoperability across different healthcare standards and institutions remains challenging due to heterogeneous data formats and ontology alignment issues. In addition, relatively few studies comprehensively address security, privacy, scalability, and maintenance of large ontology repositories. Although Artificial Intelligence and Explainable AI have recently been integrated with ontologies, their clinical validation and deployment are still limited.

Overall, the literature indicates that ontology-based healthcare systems have considerable potential to improve intelligent healthcare applications. However, future research should focus on large-scale clinical validation, standardized ontology integration, privacy-preserving frameworks, multimodal healthcare data integration, and explainable AI to facilitate reliable deployment in routine clinical practice.

### 4.6 Interpretation of performance metrics

The performance metrics presented in Table 2 were extracted directly from the original studies as reported by the respective authors. Since the reviewed studies employed different datasets, sample sizes, prediction objectives, feature sets, machine learning algorithms, validation strategies, and evaluation metrics, the reported accuracy values should not be interpreted as direct performance comparisons. Instead, these metrics are presented to provide a general overview of the effectiveness of ontology-based approaches across different healthcare applications. Therefore, the comparison is qualitative rather than quantitative and is intended to highlight overall research trends instead of ranking the performance of individual studies.

### 4.7 Evidence synthesis and research maturity

The reviewed studies represent different stages of research maturity, ranging from conceptual frameworks and prototype systems to validated implementations. Conceptual studies mainly introduce ontology architectures and semantic models without comprehensive experimental validation. Prototype systems demonstrate the feasibility of ontology-based healthcare applications using limited datasets or specific clinical scenarios. In contrast, validated implementations evaluate ontology-driven approaches using real-world datasets or clinical environments and provide stronger evidence for their effectiveness.

Although many studies report improvements in interoperability, clinical decision support, and AI-assisted healthcare, the level of evidence varies considerably. Therefore, these findings should be interpreted according to the maturity of each study rather than as universally established outcomes. Overall, the reviewed literature indicates that ontology-based approaches show promising potential for enhancing healthcare systems; however, additional large-scale clinical validation and standardized evaluation are required before widespread adoption in routine healthcare practice.

## 5 Overall problems and challenges

The problems and challenges found in the study are summarized below. These issues point out important limitations and areas that need improvement. They also give us an understanding of the gaps in current research and possible directions for future work.

- Inconsistency Among Annotators: When multiple annotators are involved, there can be significant variability in how activities are labeled, resulting in inconsistent data that can affect the performance of AR algorithms.
- Complexity of Systems: The healthcare environment is complicated, with numerous hospitals and clinics operating independently, each using different systems that do not integrate well, making management and operation challenging.
- Extraction of Semantic Elements: The system faces difficulties in accurately extracting various semantic elements, such as concepts, descriptions, and pairs of concepts and descriptions, from health care documents.
- Rising Cyber Threats: There is a notable increase in cyberattacks targeting healthcare web applications, which often contain sensitive health information. These attacks can lead to significant financial losses and privacy breaches for organizations.
- Technical Vulnerabilities: The article identifies technical issues as the most common source of security exploits in healthcare. Compared to human errors, web application vulnerabilities specifically create maximum problems and require immediate solutions to defend against potential threats.
- Inefficiencies in Data Preparation for Research: The document also highlights the burdens faced by researchers in preparing data for Institutional Review Board (IRB) approval, particularly the time-consuming process of ensuring compliance with privacy standards.
- Inadequate Understanding of Cardiac Conditions: The article points out that patients’ responses or symptoms often do not provide accurate diagnoses because many types of heart diseases exhibit similar symptoms.

### 5.1 Practical applications and remaining challenges of healthcare ontologies

The reviewed studies demonstrate that healthcare ontologies have been successfully applied in several important domains. In clinical decision support systems, ontologies facilitate standardized representation of medical knowledge, enabling improved disease diagnosis and treatment recommendations. Ontology-based semantic interoperability has enhanced information exchange among heterogeneous healthcare systems through standards such as SNOMED CT, ICD, and HL7 FHIR. Furthermore, ontologies have supported personalized medicine by integrating patient-specific clinical information with domain knowledge to enable individualized diagnosis and treatment planning. Recent studies have also combined ontologies with Artificial Intelligence, Knowledge Graphs, and Explainable AI to improve disease prediction, semantic reasoning, and clinical decision support.

Despite these advances, several important challenges remain. Many ontology-based systems have been validated only using prototype implementations or limited public datasets, with relatively few large-scale real-world clinical deployments. Semantic interoperability across different healthcare standards and institutions remains difficult because of heterogeneous data models and ontology alignment issues. In addition, ontology construction and maintenance require substantial domain expertise and continuous updates to reflect evolving medical knowledge. Privacy, security, scalability, and integration with emerging AI technologies, including Large Language Models, also remain important research challenges that require further investigation before widespread clinical adoption can be achieved.

## 6 Future enhancement

The future of using ontology and AI together in healthcare can lead to major improvements in how patients are diagnosed, treated, and cared for. Ontologies, which organize and link medical terms and concepts, help healthcare systems understand complex medical information. When combined with AI, they can analyze huge amounts of patient data to find patterns that help doctors make more accurate diagnoses and predict how diseases might develop. This could allow doctors to create personalized treatment plans based on each patient’s unique medical history, genetic background, and lifestyle. AI-enhanced ontologies also improve communication between different healthcare systems, making it easier for them to share and understand each other’s data accurately. This ensures that patient information flows seamlessly across hospitals and clinics, helping to improve the overall coordination of care. For patients, this integration can offer tailored health advice that fits their specific needs, such as lifestyle changes to prevent certain diseases. In drug research, these advanced systems can identify potential new treatments and detect side effects by connecting different pieces of medical information in new ways. AI can also scan unstructured data, like doctors’ notes, to pick out valuable insights that support better decision-making. By making it easier to manage and interpret complex healthcare data, AI-enhanced ontologies help healthcare professionals make faster, more accurate, and more personalized decisions for each patient. Overall, this combination aims to create a healthcare system that constantly improves by learning from new data. Patients get more personalized care, healthcare providers can make better decisions, and systems become more efficient. For this future to become a reality, experts from various fields will need to work together to make sure these systems are safe, ethical, and respect patient privacy.

## 7 Conclusion

In conclusion, this survey of research articles on the use of ontology in healthcare highlights both the potential and the challenges of implementing ontologies to enhance medical data integration, clinical decision-making, and personalized care. By applying the PRISMA model, we were able to systematically review and identify key problems faced in the adoption and development of healthcare ontologies. While ontologies offer significant benefits in terms of organizing complex medical knowledge and improving data interoperability across different systems, challenges such as a lack of standardization, issues with data consistency, and difficulties in keeping ontologies updated with the latest medical advancements remain prevalent. Furthermore, the integration of ontologies with emerging technologies like artificial intelligence is hindered by limitations in scalability and the complexity of handling diverse datasets. Ethical and privacy concerns also pose significant barriers, particularly in ensuring that patient data is securely managed and used responsibly. This review underscores the importance of addressing these issues in future research, with an emphasis on developing adaptable, scalable ontologies that can effectively support AI-driven healthcare systems while maintaining patient privacy and ethical standards. The findings also suggest a need for greater collaboration between healthcare providers, data scientists, and policymakers to create a cohesive framework for ontology-based healthcare systems. Moving forward, addressing these challenges will be crucial in unlocking the full potential of ontologies to improve healthcare outcomes, facilitate seamless data exchange, and support more accurate and personalized patient care.
